# Multi-Tissue Transcriptomes Yield Information on High-Altitude Adaptation and Sex-Determination in *Scutiger* cf. *sikimmensis*

**DOI:** 10.3390/genes10110873

**Published:** 2019-10-31

**Authors:** Sylvia Hofmann, Heiner Kuhl, Chitra Bahadur Baniya, Matthias Stöck

**Affiliations:** 1 Department of Conservation Biology, UFZ-Helmholtz-Centre for Environmental Research, Permoserstrasse 15, 04318 Leipzig, Germany; 2 Leibniz-Institute of Freshwater Ecology and Inland Fisheries, Müggelseedamm 301, D-12587 Berlin, Germany; kuhl@igb-berlin.de (H.K.); matthias.stoeck@igb-berlin.de (M.S.); 3 Central Department of Botany, Tribhuvan University, Kirtipur 44618, Kathmandu, Nepal; cbbaniya@gmail.com

**Keywords:** Megophryidae, RNA-seq, high-altitude-specific genes, sex determination

## Abstract

The Himalayas are one of earth’s hotspots of biodiversity. Among its many cryptic and undiscovered organisms, including vertebrates, this complex high-mountain ecosystem is expected to harbour many species with adaptations to life in high altitudes. However, modern evolutionary genomic studies in Himalayan vertebrates are still at the beginning. Moreover, in organisms, like most amphibians with relatively high DNA content, whole genome sequencing remains bioinformatically challenging and no complete nuclear genomes are available for Himalayan amphibians. Here, we present the first well-annotated multi-tissue transcriptome of a Greater Himalayan species, the lazy toad *Scutiger* cf. *sikimmensis* (Anura: Megophryidae). Applying Illumina NextSeq 500 RNAseq to six tissues, we obtained 41.32 Gb of sequences, assembled to ~111,000 unigenes, translating into 54362 known genes as annotated in seven functional databases. We tested 19 genes, known to play roles in anuran and reptile adaptation to high elevations, and potentially detected diversifying selection for two (TGS1, SENP5) in *Scutiger*. Of a list of 37 genes, we also identify 27 candidate genes for sex determination or sexual development, all of which providing the first such data for this non-model megophryid species. These transcriptomes will serve as a valuable resource for further studies on amphibian evolution in the Greater Himalaya as a biodiversity hotspot.

## 1. Introduction

The Himalayas are a distinct biogeographic eco-region with high biodiversity and endemism due to great topographic and climatic variation [1] and isolation. The uplift of Tibet and the Himalayas since about 45 million years ago (Mya), with the Greater Himalayas starting to rise presumably the earliest in the post-Eocene, or even more recently (~20–10 Mya; for a review see the supplementary in Hofmann et al. [2]) appears to have resulted in a unique assemblage of species evolved under gradual high-altitude adaptation, as already shown for several Tibetan reptiles [3] and anurans [4,5] as well as interspecies comparisons with evidence for parallel evolution [6,7,8]. However, with respect to high-altitude adaptation in the Greater Himalayas, there is still a considerable knowledge deficiency as there is a general lack of knowledge about the biodiversity of this high-mountain range, causing its relatively large number of cryptic and undiscovered species [9], even among vertebrates. Despite its poorly accessible landscapes, the fragile and vulnerable Himalayan mountain ecosystems have not been spared from land use changes, anthropogenic habitat degradation, and other increasing pressures of growing human populations as well as tourism, and the region especially suffers from global climate change [10]. The associated loss and changing conditions for biodiversity are alarming and many species may be lost before they are known [11,12].

Molecular evolutionary studies of truly Greater Himalayan animal species are extremely rare (e.g., [13]) and, particularly, genomic data are lacking for most vertebrates (e.g., a PubMed search for “genomic” and “Himalaya” yielded 72 hits, only 18 when including “vertebrates” but zero for “amphibians”). To allow future studies in population genomics and phylogenetics, for example, using sequences capture approaches and to generally close this major data gap for regional amphibians, in the present paper, we have generated a new molecular marker set based on RNAseq for an anuran species from the Greater Himalaya.

Our target species is a megophryid toad, the Sikkim lazy toad (an alternative common name is “alpine toad”). Since there is great taxonomic uncertainty among Himalayan *Scutiger* [2], we refer to this specimen as *Scutiger* cf. *sikimmensis*. Importantly, so far, no well-annotated multi-tissue nuclear transcriptome or genome of a *Scutiger* species has been characterized, although transcriptomes of the skin [14] and pooled larval tissues [15] as well as brain, testes and skin [16] of related taxa from the family Megophryidae have recently been published. Megophryidae represent the sister group of Pelobatidae [17,18], and form a highly species-diverse family of oriental anurans with a basal phylogenetic position relative to the Neobatrachia [17,19,20,21,22,23]. All species comprise stream-breeding, forest ground-dwellers with toad-like morphology [24]. Lazy toads are a characteristic faunal element of the Himalaya-Tibet-Orogen. This genus comprises 23 recognized species, most of which distributed in the Hengduan Shan and adjacent regions to the east (Province Sichuan, China). Of these 23 taxa, at least six nominal species are only known from their type localities [2,25]. The Himalayan *Scutiger* group has been shown to be an excellent model for phylogeographic and evolutionary research due to its high local endemism (associated with many, so far undescribed lineages), the extremely limited distributional ranges and adaptation to high elevations of these species and the occurrence of strictly allopatric speciation [2,26,27]. During the last 15 years, four new *Scutiger*-species have been described, highlighting the potentially great number of undiscovered taxa. However, there is poor knowledge about species’ ranges in the Greater Himalaya and adjacent regions, as well as about their taxonomic diversity [28,29] and evolution [2], and this deficit is accompanied by a lack of high-quality molecular data. 

With this study, we publish the first multi-tissue transcriptome of a high-altitude amphibian species from the Greater Himalaya and report genes known to play roles in adaptation of vertebrates to high elevations as well as in sex determination and sexual differentiation of this non-model amphibian. These data provide robust resources for future evolutionary investigations in *Scutiger*.

## 2. Materials and Methods 

### 2.1. Animal Sampling and Ethics Statement

**Figure 1:**
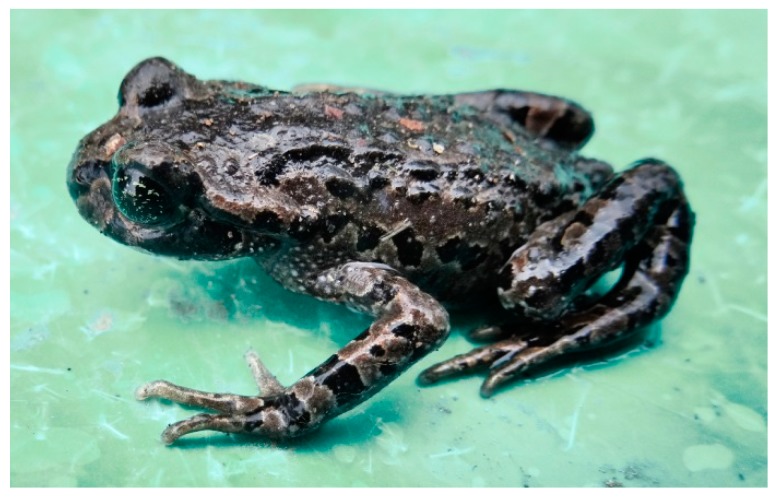
*Scutiger* cf. *sikimmensis* found in the Central Himalaya at 3289 m.

A single male *Scutiger* cf. *sikimmensis* was collected in Central Nepal, near Dunche (28.05 N, 85.26 E, 3289 m a.s.l.; Figure 1). Samples were collected in accordance with regulations for the protection of terrestrial wild animals under the permits of the Nepal expeditions of the Natural History Museum of Erfurt, Germany [30,31]. The male specimen was anesthetised in the field by immersion in tricaine methanesulfonate (MS 222; Sigma-Aldrich), sacrificed by decapitation, dissected and small pieces of organs transferred into RNAlater (Thermo Fisher), kept on ambient temperature during the time of the field work and later stored at −80 °C. 

### 2.2. RNA Extraction, cDNA Library Construction, and Illumina Sequencing

Total RNA was extracted from six different tissues (brain, heart, liver, lung, kidney, testes) and adjusted to equal concentrations. RNA integrity was assessed by RNA concentration, RIN value, 28S/18S and the fragment length distribution using an Agilent 2100 Bioanalyzer (Agilent Technologies, Inc., CA, USA). Complementary DNA (cDNA) was synthesized and sequenced by BGI (BGI-Hongkong Co., Ltd.), using the Illumina NextSeq 500 sequencing system (Illumina, San Diego, USA).

### 2.3. Filtering, De Novo Assembly, Functional Annotation and Gene Expression

The raw Illumina reads were filtered by quality and for adaptor contamination, targeting reads containing adaptor, reads with more than 5% ambiguous bases, or reads with greater than 20% of bases with quality score below 15. The remaining high-quality, clean reads were de novo assembled to transcripts using Trinity v2.0.6 [32], based on the de Bruijn graph algorithm. Transcripts were clustered using Tgicl v2.0.6 [33] to obtain sequences that could no longer be extended. The resulting sequences were defined as unigenes. They represent expressed sequences, but are not characterized sufficiently to be denoted as a complete gene. The entire unigene set was functionally annotated by matching against seven frequently used databases, including Gene Orthology (GO), Kyoto Encyclopedia of Genes and Genomes (KEGG), EuKaryotic Orthologous Groups (KOG), the non-redundant nucleotide (NT) database, the non-redundant (NR) protein database, SwissProt, and InterPro. We used Diamond v0.8.31 [34] or the BLASTx [35] algorithm with an E-value threshold of 1.0 × 10^−5^ to align unigenes to KEGG, KOG, NR, and NT and SwissProt for the annotation. Unigenes that matched to the NR database were annotated to the GO database with Blast2GOv2.5.0 [36] and classified into three categories: biological process, cellular components and molecular functions. BLAST2GO was also used to identify protein domains against the InterPro databases using the InterProScan5 v5.11-51.0 tool [37]. Candidate coding areas of these unigenes were predicted using TransDecoder v.3.0.1 (https://github.com/TransDecoder/TransDecoder) and the Pfam protein homologous sequences were searched by Blast to SwissProt and hmmscan v3.0 [38] to predict the coding region. For a coding sequence with more than two ORF, the longest one was identified as the sequence of interest. We mapped all unigenes to AnimalTFDB2.0 database and EMBOSS getorf v6.5.70 [39] to find ORF of each unigene and aligned the ORF to Transcription Factor domains using hmmsearch. 

For each sample, expression profiles were obtained by mapping clean paired-end reads back to unigenes using Bowtie2 v2.2.5 [40] with default parameters. Raw expression counts were then calculated with RSEM v1.1.20 [41]. The raw counts were normalized into FPKM values to quantify transcript levels among the different tissue samples.

Data generated in this study are publicly available from the NCBI GenBank database under the Bioproject ID PRJNA532534. All clean sequence data were deposited in the NCBI Sequence Read Archive (SRA, http://www.ncbi.nlm.nih.gov/Traces/sra/) under the accession numbers SRR9953599-SRR9953604, assembled sequences were transmitted to NCBI Transcriptome Shotgun Assembly Sequence Database (TSA, http://www.ncbi.nlm.nih.gov/genbank/tsa); the annotation dataset and statistics have been uploaded to figshare (doi 10.6084/m9.figshare.9913202).

### 2.4. Tests for Episodic Diversification in Selected Genes, Relevant for High Altitude Adaptation 

We used the 21 most prominent genes, exhibiting convergent and continuous genetic adaptation to high elevations in Ranidae and Agamidae as provided by the authors of [6] upon request for assembled transcripts and annotated coding regions; thus, no whole-transcriptome selection analysis was feasible. The unigene annotation was screened for matches to the gene symbols of altitude adapted genes. Matching unigenes were filtered from the assembled transcriptome sequences. These candidate sequences were aligned with NR protein database to manually confirm the gene assignment. Coding sequence (CDS) of the transcripts was determined with TransDecoder. CDS were aligned codon based with the sequences from Sun et al. [6] using TranslatorX [42]. Finally, we removed stop codons and all codons that contained unaligned positions in at least one species. The alignments were submitted to aBSREL analysis at www.datamonkey.org [43,44], and therein were analyzed for potential diversifying selection at all branches, comparing selection through uncorrected *p*-values in the *Nanorana*-clade to those in *Scutiger*.

### 2.5. Identification of Genes Involved in Sex Determination or Sex Differentiation

Using a list of anuran genes presumably involved in male or female sex determination and sexual differentiation [45] to identify templates, we recovered such genes from the multiple tissues in *Scutiger* and studied their tissue-specific expression. Template protein sequences from the anuran model species *Xenopus tropicalis* or *Xenopus laevis* were obtained from Xenbase [46] and aligned with BLAT [47] (−t = dnax –q = prot) against *Scutiger* transcripts assembled from RNAseq data. Matching transcripts were identified and aligned to the NR database with BLASTx [35,48]. After manual inspection, we identified and removed paralogs or unspecific sequences. Unigenes were considered homologous, if the majority of the top scoring BLASTx hits were close to the gene of interest. 

## 3. Results

### 3.1. Transcriptome

**Figure 2:**
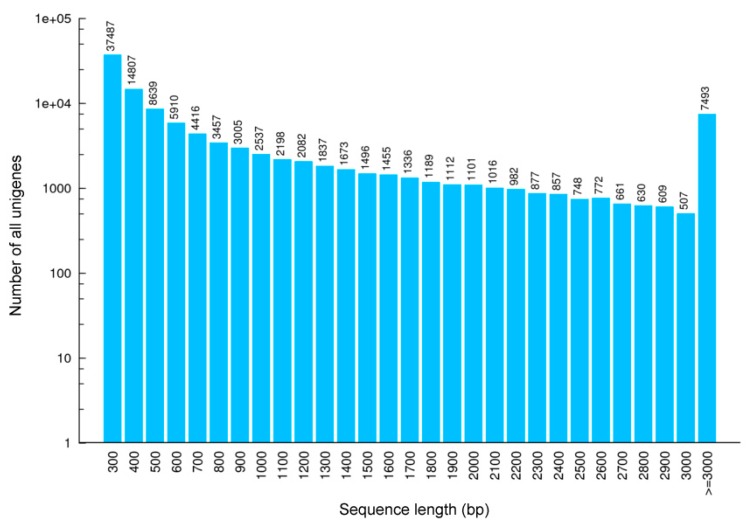
Length distribution of all unigenes based on RNA samples from different tissues of *Scutiger* cf. *sikimmensis*. The *x*-axis represents the sequence length (base pairs), while the *y*-axis represents the number of transcripts.

From separate cDNA libraries of six tissues (brain, heart, kidney, liver, lung, testis), a total of 425.4 million raw sequence data (41.32 Gb) were generated. After filtering and quality checks, 413.4 million clean reads were obtained, with an average of 68.9 million reads per tissue (Table 1). The GC-content of these clean reads ranged between 44.78% and 46.19%; 97% of all bases had a quality score of at least Q20. Over all samples, the assembly resulted in 110,889 non-redundant unigene sequences with a total length of 104,314,420 bp. Unigenes had an average length of 940 bp and a N50 of 1926 bp. A total of 103,396 unigenes ranged in length from 300 to 3000 bp (Table 2, Figure 2 and Supplementary Figure S1).

### 3.2. Functional Annotation

**Figure 3:**
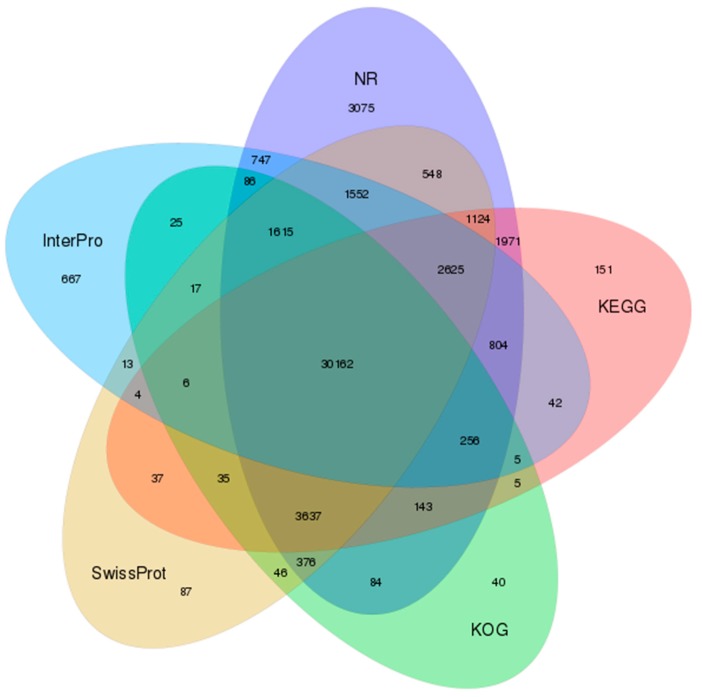
Venn diagram of shared and unique unigenes in *Scutiger* cf. *sikimmensis* among the five most informative databases used for annotation. GO = gene orthology; InterPro = integrative protein signature database; KEGG = Kyoto Encyclopedia of Genes and Genomes; KOG = EuKaryotic Orthologous Groups; NR = non-redundant protein database; NT = non-redundant nucleotide database; SwissProt = Swiss Protein Sequence Database.

Altogether, 54362 (49.02%) unigenes showed homology to entries in at least one of the seven functional databases (Figure 3, Supplementary Table S1); 11,933 unigenes were annotated by all these databases (Supplementary File S1). Most matches were obtained in the NR database, with 48805 unigenes (44.01%), of which 26,071 (23.51%) could be assigned to gene ontology terms. Regarding the biological process ontology, the most common classifications were cellular processes (13,065), metabolic processes (9499) and biological regulation (6523). The most common categories for the cellular component ontology were cellular (13,125), cell compartments (13,004) and organelles (9276). In terms of the molecular function, most common categories involved binding (13,958), catalytic activity (9871) and molecular transporter activity (1479) (Supplementary Figure S2).

The KOG functional classification revealed general function (8788), signal transduction mechanisms (8017 unigenes), posttranslational modification (3845 unigenes), unknown function (3430 unigenes) and transcription (3113) as top five categories (Supplementary Figure S3). 

We also performed a search of all unigenes against the KEGG database to identify the active biological pathways in *Scutiger* cf. *sikimmensis*. We found 41,007 unigenes that shared homology to entries, mapping to pathways related to cellular processes (8951), environmental information processing (8646), genetic information processing (6562), human diseases (20053), metabolism (13259) and organismal systems (15435). Predominantly, the unigenes were enriched in “signal transduction” (6573), followed by “global and overview maps” (5042), “cancers: overview” (4111), “infectious diseases: viral” (3725), and “immune system” (3633) categories (Figure S4).

### 3.3. Testing Potential Genetic Adaptation to High Elevations

From the *Scutiger* transcriptome-based gene set, we were able to successfully align 19 out of 21 anuran genes, exhibiting convergent and continuous genetic adaptation to high elevations in Ranidae and Agamidae (Table in Figure 1 in Sun et al. 2018 [6]). We did not recover the genes XPS and TINAGL1 from *Scutiger*. Using aBSREL, we then examined each of the 19 alignments for the occurrence of episodic diversification. We detected evidence of diversifying selection in two *Scutiger* genes (TGS1: uncorr. *p* = 0.0339, corr. *p* = 0.2373; SENP5: uncorr. *p* = 0.0053, corr. *p* = 0.0475). In all analyses, the test statistics confirmed the selection shown by Sun et al. in *Nanorana*, as reported by these authors (Supplementary File S2: note that corrected *p-*values are provided therein as “Test *p-*value”) [6].

### 3.4. Genes Related to Sex Determination or Sex Differentiation and Their Expression Levels

Of a list of 37 genes from the clawed frog genomes, about 73% (27) had significant blast hits in the *Scutiger* transcriptomes, all of which provide the first sequence data for these genes in this non-model anuran species. In comparison to the length of *Xenopus* mRNAs, which were used as BLAST templates, the majority of the Himalayan *Scutiger* unigenes is shorter (between 4% and 98%) and may thus represent fragments. However, unigenes are also up to 3.5 times longer, which may be indicative of insertions (Table 3). The majority (∼49%) of the target genes were expressed in all tissues and only ∼5% were expressed solely in a single one. 

## 4. Discussion

We here report the first well-annotated transcriptome data of a high-altitude amphibian taxon from the Greater Himalaya, based on RNAseq of multiple tissues. Our work, based on a male *Scutiger* cf. *sikimmensis* from central Nepal, provides a transcriptome of a representative species from a branch of the amphibian tree of life (Pelobatoidea of some authors [49]; Mesobatrachia of others [50], currently Megophryidae [27]), from which no complete genomes have been sequenced. Although genomic approaches have been used [14,15], to our knowledge no annotated multi-tissue transcriptome has been published for the genus *Scutiger*. Our study is mostly descriptive, but it has yielded novel discoveries and represents an important turning point for genomic studies in megophryd anurans. 

### 4.1. Indications for Potential Genetic Adaptation to High Elevations in Scutiger

Sun et al. [6] used comparative transcriptomic analyses to prove that amphibian and reptile populations, occurring at different altitudes around the Tibetan Plateau, show parallel evolution. They have provided evidence for convergent and continuous genetic adaptation to high elevations in taxa as distant as Anura (Ranidae) and Sauropsida (Agamidae), in which genes with related functions, especially DNA-repair and energy metabolism pathways, exhibit evidence for rapid change and continuous positive selection with increasing elevations. These data let us assume that a similar genomic high-elevation-selection-syndrome might be detectable in *Scutiger*, sampled in 3289 m above sea level (Methods). Indeed, in two out of 19 key genes [6], we have detected diversifying selection and thus potentially positive selection in the *Scutiger* transcriptome as well. In another three of these key genes (PGS: uncorr. *p* = 0.180, corr. *p* = 1.000; OLFM4: uncorr. *p* = 0.0818, corr. *p* = 0.6542; PPIL2: uncorr. *p* = 0.185, corr. *p* = 1.000) we obtained uncorrected *p*-values close to significance level, possibly indicating weak signs of adaptive molecular evolution. However, the analyses of only a single *Scutiger*-transcriptome (data were unobtainable from a whole radiation of species or an altitudinal gradient as available for Ranidae [6]) limits our approach and did not allow for further comparative tests. Therefore, future research with multiple species and a greater scale of altitudinal variation might yield additional evidence for genes adapted to the life in high altitudes in lazy toads. This seems especially justified since similar traits have been studied extensively in other vertebrates, such as mammals including humans [51].

### 4.2. Expression of Genes Involved in Sex Determination or Sex Differentiation

As a second application, we used the transcriptomes of *Scutiger* cf. *sikimmensis* to search for candidate genes, known for their role in sex determination or sex differentiation in other vertebrates (e.g., [45]). Most, if not all, anurans exhibit genetic sex determination [52,53,54] and generally exhibit sex-biased gene expression, depending on phenotypic sex [55]. As most anurans [56], to our knowledge, *Scutiger* exhibits homomorphic sex chromosomes and nothing is known about sex determination in *Scutiger*.

Of the 27 genes with potential sex roles, detected in *Scutiger* cf. *sikimmensis,* two tissues, the brain (32%) and the testes (22%), exhibited the highest numbers of highly expressed genes, as typical of sex determination or differentiation genes. About a quarter of the relevant 37 genes have remained uncharacterized, presumably, since their levels of expression was below the threshold to be assembled into unigenes or might be expressed only during earlier developmental stages or exclusively in females.

## 5. Conclusions

Overall, our data provide the first well-annotated multi-tissue transcriptomic resource for a Himalayan amphibian of the genus *Scutiger*. This transcriptome is available for further studies on evolution and adaptation in Himalayan high-altitude vertebrate species.

## Figures and Tables

**Table 1:** Summary of transcriptome sequencing based on RNA samples from six different tissues.

	Brain	Heart	Kidney	Liver	Lung	Testis
Number of raw reads	62,192,592	73,368,700	66,429,410	64,241,792	87,261,442	71,858,834
Clean Reads	60,440,140	70,845,324	64,422,228	63,141,364	85,147,870	69,426,236
Clean Bases (Gb)	6.04	7.08	6.44	6.31	8.52	6.94
Clean Reads Q20 (%)	97.08	97.32	97.11	96.93	97.06	96.84
Clean Reads Q30 (%)	93.51	93.86	93.55	93.23	93.44	93.00
GC Clean Reads (%)	44.78	45.53	46.19	45.65	45.48	45.90

*N* = reads containing >5% unknown nt; Q20 = reads with base call accuracy of 99%; Q30 = reads with base call accuracy of 99.9%.

**Table 2:** Summary of assembled unigenes per tissue.

	Brain	Heart	Kidney	Liver	Lung	Testis	All Unigenes
Number of unigenes	63,189	50,037	67,145	44,915	62,363	53,203	110,889
Total length	57,057,129	40,054,175	51,103,731	34,377,766	49,335,973	43,320,879	104,314,420
Mean length	902	800	761	765	791	814	940
N50 length	1740	1448	1301	1310	1426	1461	1926
GC content %	44.51	44.73	45.75	44.20	44.34	44.55	44.70
300–500 bp	34,328 (54.33%)	28,492 (56.94%)	38,309 (57.05%)	25,402 (56.56%)	35,569 (57.04%)	29,194 (54.87%)	60,933 (54.95%)
600–1000 bp	11,604 (18.36%)	9438 (18.86%)	13,503 (20.11%)	8933 (19.89%)	11,547 (18.52%)	10,152 (19.08%)	19,325 (17.43%)
1100–2000 bp	9286 (14.70%)	7310 (14.61%)	9800 (14.60%)	6744 (15.02%)	9297 (14.91%)	8476 (15.93%)	15,479 (13.96%)
2100–3000	4476 (7.08%)	2970 (5.94%)	3576 (5.33%)	2651 (5.90%)	3851 (6.18%)	3680 (6.92%)	7659 (6.91%)
≥ 3000 bp	3495 (5.53%)	1827 (3.65%)	1957 (2.91%)	1185 (2.64%)	2099 (3.37%)	1701 (3.20%)	7493 (6.76%)

N50 length = weighted median statistic that 50% of the total length is contained in unigenes that are equal to or larger than this value.

**Table 3:** Sex-determining and sex differentiation gene inventory and homologous *Scutiger* cf. *sikimmensis* transcripts. Gene expression is calculated as FPKM based on RNA-seq data from six tissues; the highest gene expression level per gene over all tissues is indicated in bold. CL = cluster of several unigenes.

*Xenopus* Protein Sequence					*S.* cf. *sikimmensis* Transcript			Expression in Tissue (FPKM)					
Gene	Organism	Accession No.	mRNA length, nt	Protein length, amino acids	unigene	E-Value	Length, nt	Brain	Heart	Kidney	Liver	Lung	Testis
ALDH1A2	*X. tropicalis*	AAI57514.1	633	211	Unigene17092_All	0.00	2208	3.88	1.33	1.25	1.56	6.60	**6.97**
ALDH1A3	*X. tropicalis*	XP_002939310.1	4056	512	Unigene17754_All	0.00	2443	3.87	10.22	**13.05**	6.00	7.19	6.42
AR	*X. tropicalis*	XP_002941888.2	3497	788	Unigene59002_All	2.00 × 10^−33^	247	0.00	**0.64**	0.00	0.39	0.00	0.00
AR	*X. tropicalis*	XP_002941888.2	3497	788	Unigene61378_All	9.00 × 10^−55^	247	0.00	**1.27**	0.00	0.00	0.00	0.00
CTNNB1	*X. tropicalis*	NP_001016958.1	3382	781	Unigene8277_All	0.00	3640	**182.62**	88.56	60.64	29.71	57.76	83.16
CTNNB1	*X. tropicalis*	NP_001016958.1	3382	781	Unigene10320_All	1.00 × 10^−4^	815	7.56	1.32	2.78	1.17	3.02	1.13
CXCR4B	*X. laevis*	NP_001080681.1	2115	358	Unigene12380_All	0.00	3085	8.85	3.18	5.29	11.14	**104.79**	2.93
CYP26A1	*X. tropicalis*	AAI71087.1	1458	492	Unigene67459_All	0.00	1560	0.45	0.00	0.00	0.00	0.00	**6.07**
CYP26B1	*X. tropicalis*	AAI35552.1	6137	511	Unigene661_All	0.00	3759	**5.93**	4.97	0.07	0.43	0.14	0.17
CYP26C1	*X.tropicalis*	XP_002939137.2	4692	533	Unigene43005_All	0.00	2481	**6.28**	0.07	0.03	0.03	0.13	0.03
DHH	*X. tropicalis*	NM_001097169.1	4372	396	Unigene27079_All	2.00 × 10^−130^	1359	0.53	0.09	2.18	0.10	0.99	**4.17**
DHH	*X. tropicalis*	NM_001097169.1	4372	396	Unigene63854_All	5.00 × 10^−71^	366	0.75	0.00	0.90	0.23	0.69	1.55
DMRT1	*X. tropicalis*	XP_012808036.1	1011	337	Unigene30377_All	2.00 × 10^−33^	312	0.61	0.47	0.00	0.00	**1.06**	0.00
FGF9	*X. tropicalis*	XP_002938621.1	624	208	Unigene13573_All	0.00	933	**1.92**	0.00	0.23	0.16	0.00	0.60
FOXL2	*X. tropicalis*	XP_004917868.1	978	326	Unigene52644_All	1.00 × 10^−19^	271	**1.10**	0.00	0.00	0.00	0.00	0.00
GATA-4	*X. tropicalis*	NP_001016949.1	1599	394	CL10318.Contig1_All	5.00 × 10^−178^	4385	0.00	**11.62**	0.00	3.04	0.00	0.00
GATA-4	*X. tropicalis*	NP_001016949.1	1599	394	CL10318.Contig2_All	0.00	4283	0.04	**12.70**	0.03	3.56	0.02	0.29
HHIP	*X. tropicalis*	NM_001007190.1	2717	669	Unigene21373_All	0.00	861	0.47	0.00	0.17	**3.31**	1.61	0.08
HHIP	*X. tropicalis*	NM_001007190.1	2717	669	Unigene57656_All	3.00 × 10^−93^	448	0.00	0.00	0.00	0.72	0.41	0.00
LRPPRC	*X. tropicalis*	NP_001039203.1	4347	1391	Unigene19789_All	0.00	4425	16.93	23.44	18.77	9.94	9.55	**31.14**
NR0B1	*X. tropicalis*	XP_002933661.1	834	278	CL4129.Contig1_All	1.00 × 10^−122^	772	0.00	0.33	0.00	**1.61**	0.07	0.00
NR0B1	*X. tropicalis*	XP_002933661.1	834	278	CL4129.Contig2_All	4.00 × 10^−179^	1108	1.11	0.00	0.00	**11.54**	0.00	0.00
NR0B1	*X. tropicalis*	XP_002933661.1	834	278	Unigene53513_All	4.00 × 10^−46^	540	0.26	0.00	0.00	**0.89**	0.00	0.00
PDGFa	*X. tropicalis*	NM_001170497.1	1574	660	Unigene15273_All	2.00 × 10^−116^	1671	**4.89**	0.87	0.80	0.79	0.25	2.46
PDGFb	*X. tropicalis*	AAI60575.1	2140	240	Unigene19730_All	2.00 × 10^−81^	1802	5.74	3.99	3.56	5.27	**14.62**	1.19
PTCH2	*X. tropicalis*	XP_002937129.2	6786	1423	Unigene8457_All	0.00	2946	**3.35**	0.12	0.86	0.12	0.41	1.12
RSPO-1	*X. tropicalis*	NP_001121500.1	1946	257	Unigene19575_All	3.00 × 10^−118^	1913	1.27	0.32	2.84	2.10	1.69	**3.29**
SOX10	*X. tropicalis*	NP_001093691.1	2895	436	Unigene39328_All	0.00	3641	**15.94**	0.28	0.04	0.06	0.14	0.16
SOX8	*X. tropicalis*	XP_002932315.2	2389	466	Unigene17029_All	0.00	3334	**7.16**	1.44	0.55	0.29	0.41	0.40
SOX9	*X. tropicalis*	AAT72000.1	2538	482	Unigene17030_All	0.00	1955	**10.67**	1.20	1.14	1.73	0.00	0.34
SRD5A1	*X. tropicalis*	NP_001006841.1	1537	257	CL1692.Contig1_All	9.00 × 10^−5^	1964	**2.31**	0.92	2.07	1.12	0.15	1.51
SRD5A1	*X. tropicalis*	NP_001006841.1	1537	257	Unigene12175_All	1.00 × 10^−125^	1559	2.33	0.70	3.87	5.56	0.79	**5.99**
SRD5A3	*X. laevis*	AAH42255.1	957	319	CL10211.Contig1_All	1.00 × 10^−32^	295	**0.79**	0.00	0.00	0.00	0.63	0.29
SRD5A3	*X. laevis*	AAH42255.1	957	319	CL10211.Contig2_All	1.00 × 10^−95^	931	3.37	3.70	3.26	1.48	**4.23**	3.69
WNT4	*X. tropicalis*	NP_001239015.1	1962	351	Unigene64864_All	2.00 × 10^−44^	216	0.00	0.00	0.00	0.00	0.00	**1.34**
WT1	*X. tropicalis*	NP_001135625.1	6193	413	CL1216.Contig4_All	0.00	3364	0.86	1.28	2.58	0.55	6.20	**4.09**
WT1	*X. tropicalis*	NP_001135625.1	6193	413	CL4987.Contig2_All	3.00 × 10^−33^	308	0.00	0.00	**1.39**	0.00	0.00	0.28
WT1	*X. tropicalis*	NP_001135625.1	6193	413	Unigene71862_All	1.00 × 10^−26^	264	0.00	0.58	**1.01**	0.00	0.00	0.00

## Supplementary Materials

The following are available online at https://www.mdpi.com/2073-4425/10/11/873/s1, Figure S1: Frequency length distribution of unigenes in the *Scutiger* cf. *sikimmensis* transcriptome, Figure S2: Gene ontology classification of *Scutiger* cf. *sikimmensis* transcriptome unigenes, Figure S3: KOG functional classification of *Scutiger* cf. *sikimmensis* transcriptome unigenes, Figure S4: Functional distributions of *Scutiger* cf. *sikimmensis* transcriptome unigenes annotated by KEGG. Table S1: Annotation summary for the transcriptomes of *Scutiger* cf. *sikimmensis*, File S1: Annotation results of unigenes from five databases, File S2: Alignments and test statistics for 19 genes, potentially exhibiting diversifying selection in *Scutiger* cf. *sikimmensis* in comparison with ranid frogs [6].
