# Supplementary material for: Multi-Tissue Transcriptomes Yield Information on High-Altitude Adaptation and Sex-Determination in Scutiger cf. sikimmensis
Source: Genes (Basel). 2019 Oct 31;10(11):873. doi: 10.3390/genes10110873 (PMC6895926; doi:10.3390/genes10110873)
Supplement: Supplementary file 1 [file genes-10-00873-s001.zip › genes-618685-supplementary/genes-618685sup/FigS1-S4_TableS1.docx]

**Supplementary**

| 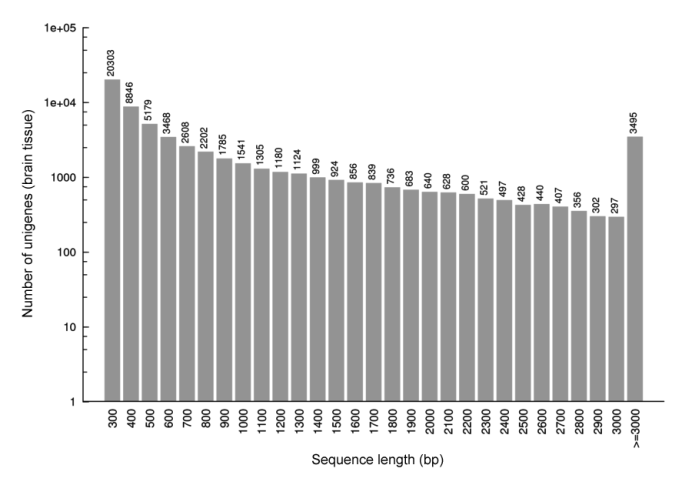 | 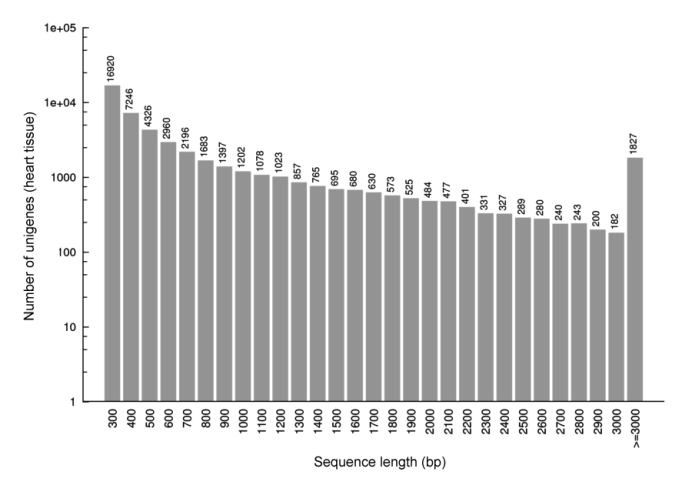 |
| --- | --- |
| 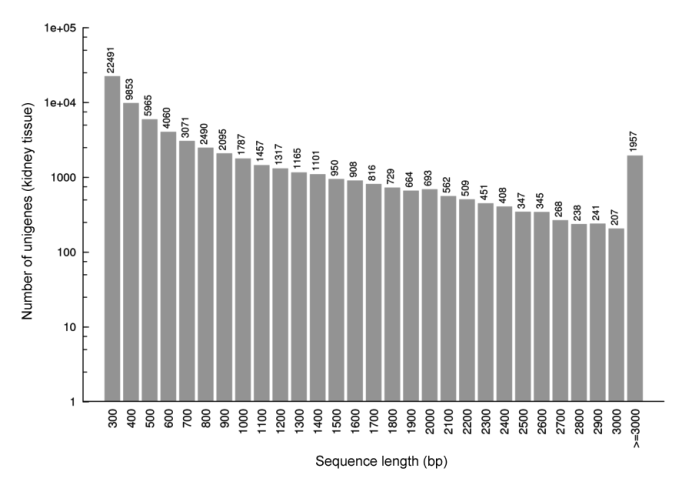 | 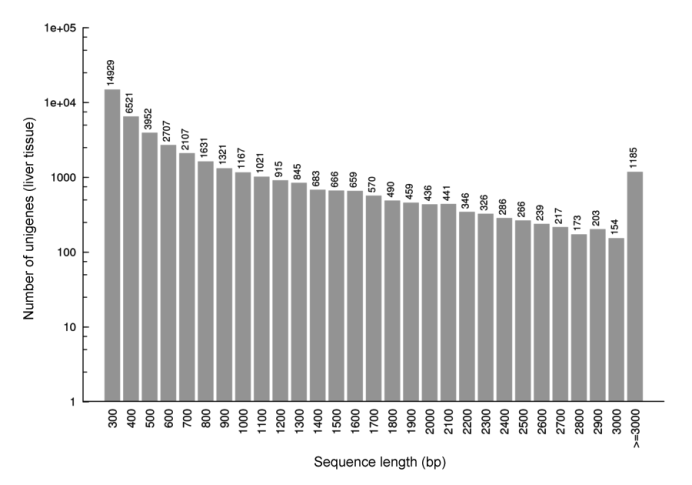 |
| 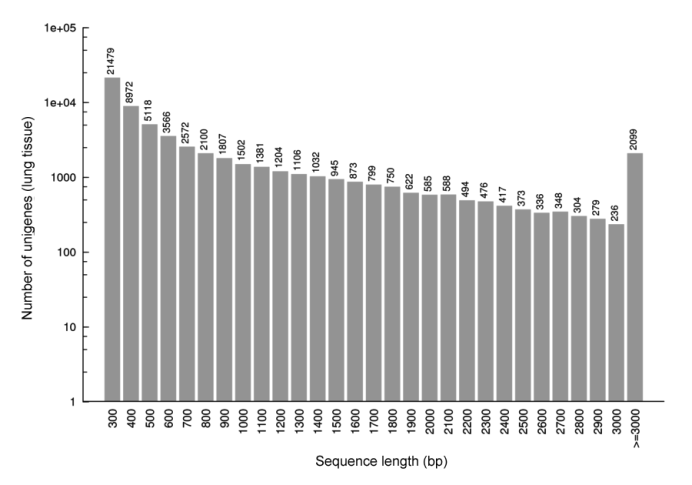 | 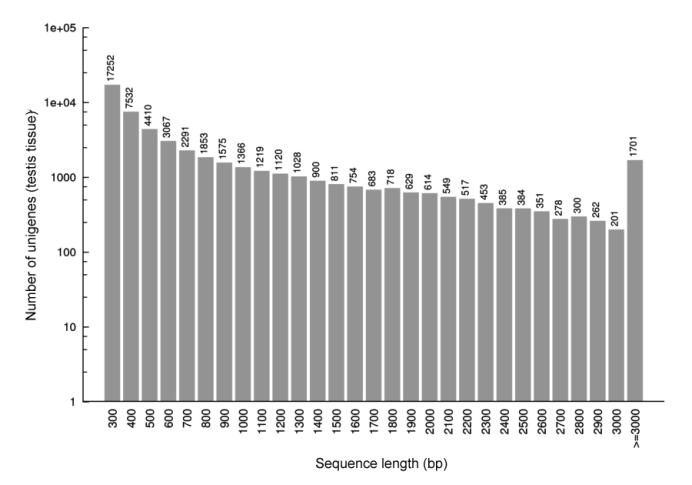 |

**Figure 1.** Frequency length distribution of unigenes in the *Scutiger* cf. *sikimmensis* transcriptome for the respective tissue (above: brain, heart; middle: kidney, liver; below: lung, testis).


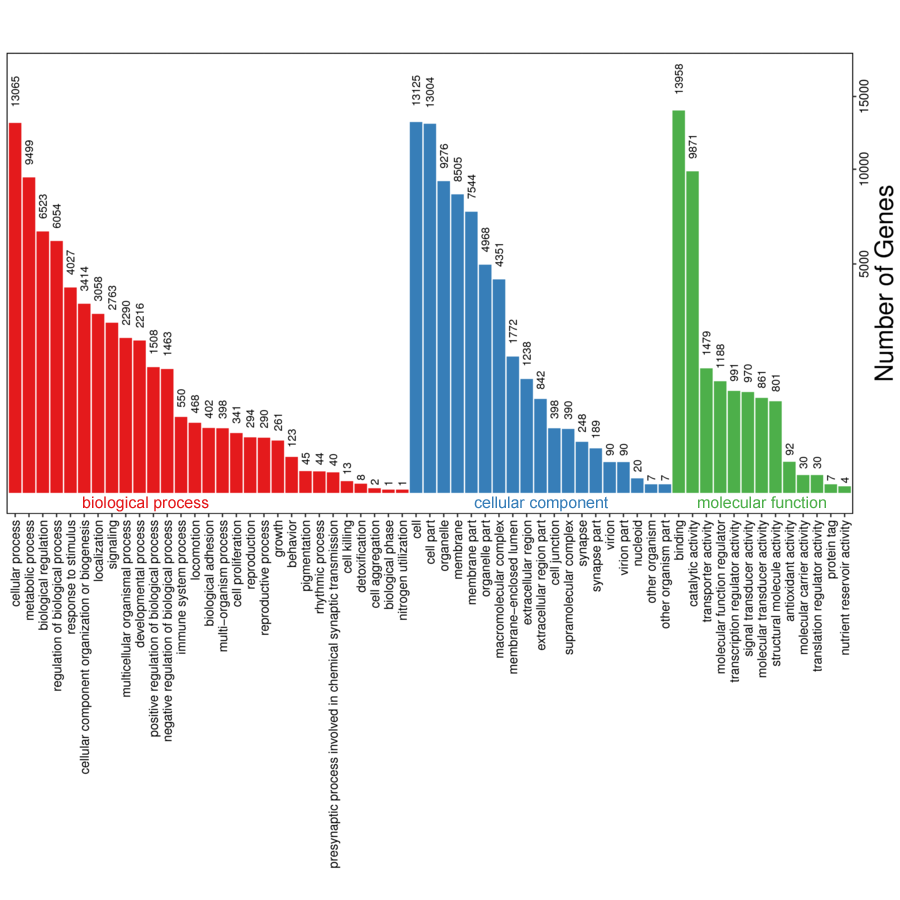


**Figure S2.** Gene ontology classification of *Scutiger* cf. *sikimmensis* transcriptome unigenes.


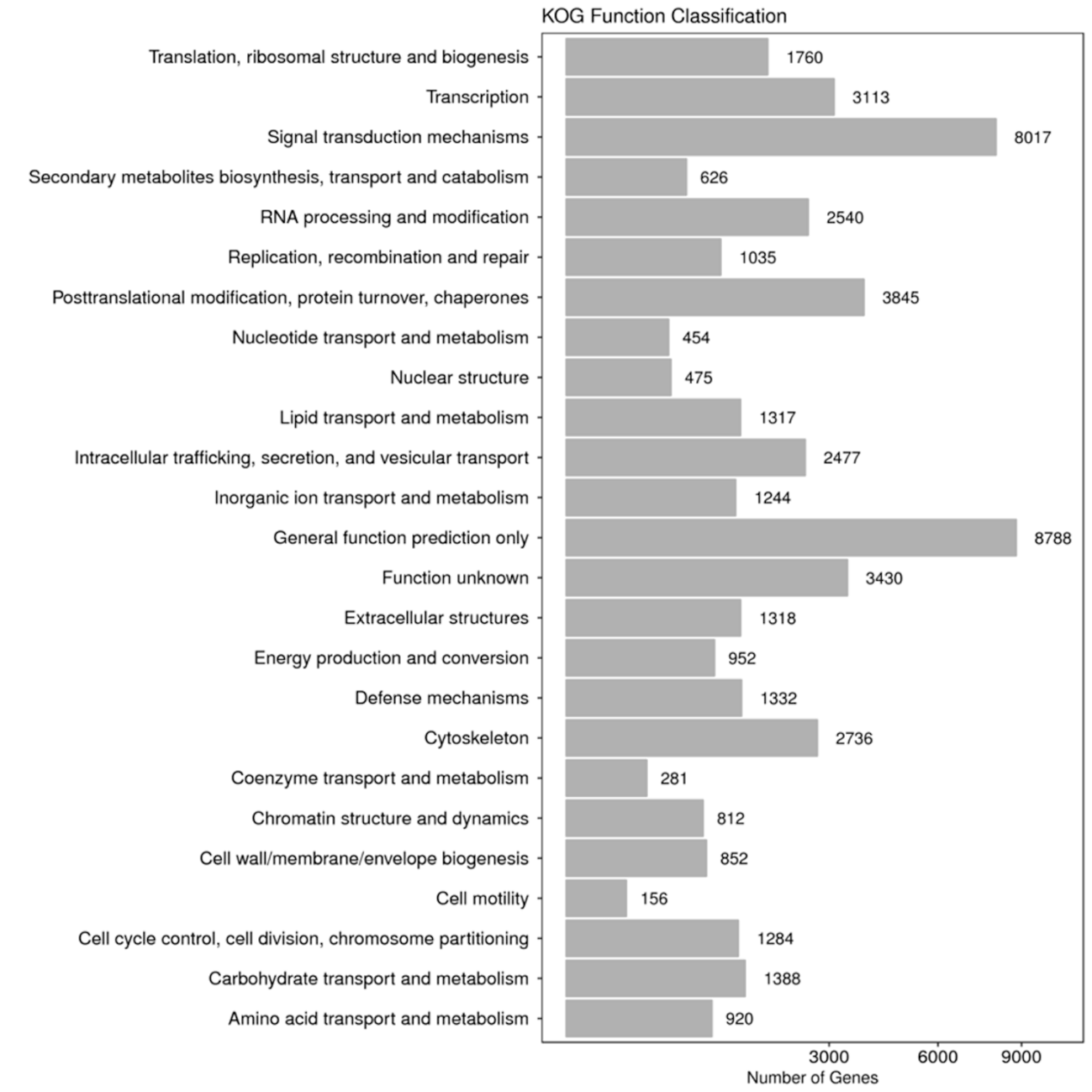


**Figure S3.** KOG functional classification of *Scutiger* cf. *sikimmensis* transcriptome unigenes.

**
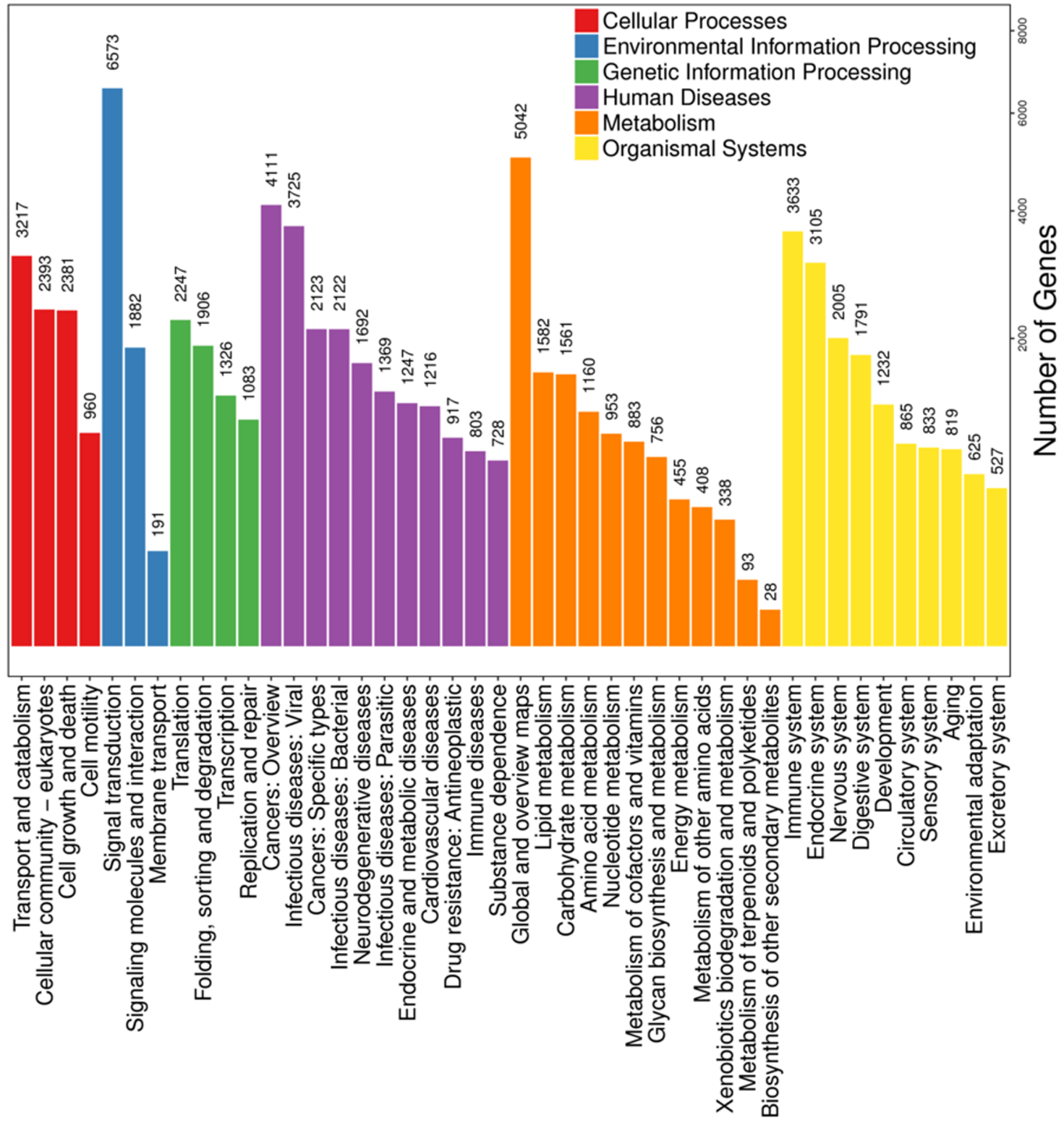
**

**Figure S4.** Functional distributions of *Scutiger* cf. *sikimmensis* transcriptome unigenes annotated by KEGG.

**Table S1.** Annotation summary for the transcriptomes of *Scutiger* cf. *sikimmensis*. Intersection = the number of unigenes, which were annotated by all seven functional databases; Overall = number of unigenes which were annotated by any of the seven functional databases (see also Figure 2).

|  | ***Total*** | ***GO*** | ***InterPro*** | ***KEGG*** | ***KOG*** | ***NR*** | ***NT*** | ***SwissProt*** | ***Intersection*** | ***Overall*** |
| --- | --- | --- | --- | --- | --- | --- | --- | --- | --- | --- |
| Number of unigenes | 110,889 | 26,071 | 38,626 | 41,007 | 36,538 | 48,805 | 27,732 | 41,884 | 11,933 | 54,362 |
| Percentage | 100% | 23.51% | 34.83% | 36.98% | 32.95% | 44.01% | 25.01% | 37.77% | 10.76% | 49.02% |
